# Self-medication with over-the-counter drugs and complementary medications in South Australia's elderly population

**DOI:** 10.1186/1472-6882-9-42

**Published:** 2009-11-11

**Authors:** Lynn Yeen Goh, Agnes I Vitry, Susan J Semple, Adrian Esterman, Mary A Luszcz

**Affiliations:** 1 Sansom Institute, University of South Australia, Adelaide, South Australia, Australia; 2 School of Nursing and Midwifery, University of South Australia, Adelaide, South Australia, Australia; 3 School of Psychology and Flinders Center for Aging Studies, Flinders University, South Australia, Australia

## Abstract

**Background:**

A number of surveys have examined use of complementary and alternative medicines (CAM) in Australia. However, there are limited Australian data on use of CAM and over-the-counter (OTC) medicines in the elderly population. The main aims of this study were to examine self-medication practices with CAM and OTC medicines among older Australians and variables associated with their use.

**Methods:**

The Australian Longitudinal Study of Ageing (ALSA) is an ongoing multidisciplinary prospective study of the older population which commenced in 1992 in South Australia. Data collected in 4 waves of ALSA between 1992 and 2004 were used in this study with a baseline sample of 2087 adults aged 65 years and over, living in the community or residential aged care. OTC medicines were classified according to the World Health Organization Anatomical Therapeutic Chemical (ATC) classification. CAM were classified according a modified version of the classification adopted by the Therapeutics Goods Administration (TGA) in Australia.

**Results:**

The prevalence of CAM or OTC use ranged from 17.7% in 2000-2001 to 35.5% in 2003-2004. The top classes of CAM and OTC medicines used remained relatively constant over the study period. The most frequent classes of CAM used were vitamins and minerals, herbal medicines and nutritional supplements while the most commonly used OTC were analgesics, laxatives and low dose aspirin. Females and those of younger age were more likely to be CAM users but no variable was associated with OTC use.

**Conclusion:**

Participants seemed to self-medicate in accordance with approved indications, suggesting they were informed consumers, actively looking after their own health. However, use of analgesics and aspirin are associated with an increased risk of adverse drug events in the elderly. Future work should examine how self-medication contributes to polypharmacy and increases the risk of adverse drug reactions.

## Background

In Australia, the proportion of adults over the age of 65 in 2005 was 13% and this figure is expected to more than double by 2051. South Australia has the highest percentage of people aged 65 and over of all Australian states and territories, and this age group is expected to make up 26.5% of the state's population by 2031[1].

Increasing age is associated with increased prevalence of chronic medical conditions [2], a higher number of medicines used, and a higher demand for all medical services, including alternative services [3,4]. High levels of self-medication practices with over-the-counter (OTC) medicines and complementary and alternative medications (CAM) have been reported in Australia and comparable countries [5-9]. The most extensive survey of the South Australian population in 2004 found that CAMs were used by 52.2% of the respondents with the greatest use in better-educated, higher income, women in the 25-44 years of age group living in a metropolitan area [6]. Use of OTC drugs and CAM in the elderly is of particular interest because several variables associated with old age such as polypharmacy, multiple comorbid illnesses and physiological changes, can increase the risk of adverse drug reactions. Most research on use of CAM and OTC medicines has focused on groups of people with specific chronic conditions. The use of CAM and OTC drugs in the general elderly population remains under-studied internationally.

The Australian Longitudinal Study of Ageing (ALSA) is an ongoing multidisciplinary prospective study of the older population which commenced in 1992 in South Australia. People initially recruited in the study were re-interviewed at multiple time points and ten waves of data collection have been carried out to date. An earlier assessment showed that, at baseline, more than one quarter of the participants were taking at least five medications concurrently [10]. One third of the participants were using non-prescription medications in combination with prescribed medicines. Our study aimed to examine self-medication practices among older Australians, to ascertain the most commonly used non-prescription medications and variables shown in previous research to be associated with their use (i.e. age, gender, education, income and health).

## Methods

ALSA data were used in this study. Methods for inclusion of subjects and survey tools have been described in detail previously [10]. In brief, subjects aged 65 years and over were randomly drawn from the South Australian Electoral Roll. The sample was stratified by age and gender. In this report, 'age' is dichotomized as 65-79 years or more than 80 years of age. Categories for the other background variables of interest are shown in Table 1. Comprehensive personal interviews were performed in wave 1 (1992-1993), wave 3 (1994-1995), wave 6 (2000-2001) and wave 7 (2003-2004). Participants were asked about all medicines that they had taken or were supposed to take in the last two weeks. For each medicine, the drug name, dose, duration of treatment, indication for use, whether it was prescribed by a doctor and the reason for use was asked. Reasons for use were grouped a posteriori into categories specific to the type of products. Participants were also asked to show the medicine container to the interviewer.

**Table 1:** Characteristics of participants at waves 1, 3, 6 and 7

	1992-1993		1994-1995		2000-2001		2003-2004	
	Wave 1	N = 2087	Wave 3	N = 1679	Wave 6	N = 791	Wave 7	N = 487
	
Variable	N	%	N	%	N	%	N	%
**Age (years)**								
65-79	1224	58.6	893	53.2	216	27.3	47	9.7
80+	863	41.4	786	46.8	575	72.7	440	90.3
**Gender**								
Female	1031	49.4	854	50.9	456	57.6	303	62.2
Male	1056	50.6	825	49.1	335	42.4	184	37.8
**Education** **(Age when left school)**								
<14 years	336	16.3	262	15.4	105	13.3	65	13.4
≥ 14 years	1725	83.7	1399	84.6	684	86.7	420	86.6
Missing	26		18		2		2	
**Tertiary education**								
Yes	700	33.9	570	34.2	278	35.2	177	36.5
No	1367	66.1	1096	65.8	512	64.8	308	63.5
Missing	20		13		1		2	
**Income Level**								
<$12,000	686	35.5	559	40.9	165	30.3	116	35.6
$12,000 - $30,000	1083	56.1	719	52.6	312	57.4	146	44.8
>$30,000	161	8.3	90	6.6	67	12.3	64	19.6
Missing	157		311		247		161	
**Self-rated health**								
Excellent	191	9.2	129	8.2	64	9.8	16	3.9
Very Good	599	28.8	410	25.9	181	27.7	85	20.5
Good	633	30.4	534	33.8	224	34.3	171	41.2
Fair	477	22.9	403	25.5	147	22.5	113	27.2
Poor	181	8.7	106	6.7	37	5.7	30	7.2
Missing	6		97		138		72	

Missing data are not included in the calculation of the percentages

OTC medicines were classified according to the World Health Organization Anatomical Therapeutic Chemical (ATC classification). Self-reported OTC medicines belonged to ten ATC categories: analgesics, laxatives, antithrombotic agents, antacids, cough and cold preparations, antihistamines, dermatologicals, throat preparations, nasal preparations and antidiarrhoeals. CAM were classified according to the classification adopted by the Therapeutics Goods Administration (TGA) in Australia [11]. The TGA classification was modified to exclude aromatherapy products (no reports) and to include two additional categories, probiotics and combination products to account for those medicines containing multiple ingredients that fell into different classes of CAM. The final CAM classification included seven categories: herbal medicines, traditional medicines (products identified as those used in a specific traditional medical system such as Traditional Chinese Medicine), vitamins and minerals, nutritional supplements, homeopathic medicines, probiotics and combination products.

The most common variables associated with use of CAM or OTC medicines were identified through a literature review of relevant studies published between 1985 and 2007. Relevant variables available in the ALSA dataset included age, gender, education level, income level and self-rated health. Pearson's chi-square tests were carried out to determine the statistical significance of the association between use of CAM and OTC medicines and the variables selected. Data were analysed using the Statistical Package for Social Sciences (SPSS) version 14.0. This study was approved by the Ethics Committee of the University of South Australia.

## Results

### Characteristics of participants

A baseline sample of 2087 participants was interviewed in wave 1 with 1679, 791 and 487 participants re-interviewed in waves 3, 6 and 7 respectively. Attrition was mainly due to mortality (58.7% at wave 7) and refusal to participate (13.6% at wave 7), the latter most often due to frailty and ill-health.

At baseline there were approximately equal numbers of men and women and 84% reported leaving school at age fourteen or over. The percentage of female participants increased from 49.4% in 1992-1993 to 62.2% in 2003-2004. The percentage of participants over the age of 80 years increased from 41.4% in the 12-year period between 1992-1993 to 90.3% in 2003-2004 [Table 1]. Across the waves, the majority of participants rated their own health as good, very good or excellent and about of third of participants rated their health as fair or poor [Table 1].

### Prevalence of self-medication

The range of participants reporting using either at least one CAM or one OTC medicine varied from 19.4% in 1992-1993 to 35.5% in 2003-2004 [Table 2]. Correspondingly, when examined separately, the use of CAM and OTC medicines varied from 12.8% to 17.0%, and 8.6% to 24.2% respectively over this time period.

**Table 2:** Number of participants using either CAM and OTC medicines

	1992-1993		1994-1995		2000-2001		2003-2004	
	Wave 1	N = 2087	Wave 3	N = 1679	Wave 6	N = 791	Wave 7	N = 487
	
Variable	N	%	N	%	N	%	N	%
**CAM or OTC^1^**	404	19.4	460	27.4	140	17.7	173	35.5
**OTC only**	268	12.8	278	16.6	79	10.0	83	17.0
**CAM only**	180	8.6	241	14.4	71	9.0	118	24.2

^1 ^Users of either one CAM or OTC medicine.

Top classes of CAM and OTC use were relatively constant across the waves [Table 3]. Vitamins and minerals were the most commonly used CAM products at all waves (5.4% to 14.0%). This was followed by herbal medicines (2.4% to 5.5%) and nutritional supplements (1.6% to 7.6%). Most of the combination products (2.1% to 5.1%) included different herbal combinations. Multivitamins and vitamin C were the most commonly used vitamins. Garlic, celery and gingko biloba were the most commonly used herbal medicines and cod liver oil was the most popular nutritional supplement [Table 4].

**Table 3:** Prevalence of use of CAM and OTC medicines

		1992-1993		1994-1995		2000-2001		2003-2004	
		Wave 1	N = 2087	Wave 3	N = 1679	Wave 6	N = 791	Wave 7	N = 487
		
	Classes of CAM/OTC	N	%	N	%	N	%	N	%
**CAM**	Vitamins & minerals	113	5.4	155	9.2	53	6.7	68	14.0
	Herbal medicines	50	2.4	69	4.1	23	2.9	27	5.5
	Nutritional supplements	34	1.6	54	3.2	26	3.3	37	7.6
	Homeopathic medicines	4	0.2	5	0.3	0	0.0	1	0.2
	Probiotics	3	0.1	4	0.2	0	0.0	0	0.0
	Combination products	43	2.1	49	2.9	9	1.1	25	5.1

**OTC**	Analgesics	112	5.4	127	7.6	44	5.6	43	8.8
	Laxatives	89	4.3	76	4.5	16	2.0	25	5.1
	Antithrombotic agents	34	1.6	43	2.6	17	2.1	10	2.1
	Antacids	19	0.9	14	0.8	1	0.1	6	1.2
	Cough & cold preps	16	0.8	18	1.1	0	0.0	5	1.0
	Antihistamines	7	0.3	4	0.2	2	0.3	1	0.2
	Dermatologicals	3	0.1	4	0.2	1	0.1	0	0.0
	Throat preparations	4	0.2	0	0.0	0	0.0	0	0.0
	Nasal preparations	2	0.1	5	0.3	0	0.0	0	0.0
	Antidiarrhoeals	2	0.1	1	0.1	0	0.0	0	0.0

	Other^1^	11	0.5	16	1.0	4	0.5	4	0.8
	Unknown^2^	11	0.5	10	0.6	3	0.4	12	2.5

The percentages show the proportions of participants reporting to use at least one of the classes of CAM or OTC medicines

^1 ^Other CAM and OTC classes that were less commonly reported

^2 ^Non-prescription medicines of unknown ingredients that could not be classified

**Table 4:** Most commonly used CAM

	1992-1993		1994-1995		2000-2001		2003-2004	
	Wave 1	N = 180	Wave 3	N = 241	Wave 6	N = 71	Wave 7	N = 118
	
CAM	N	%	N	%	N	%	N	%
**Vitamins and minerals**								
Multivitamins	40	22.2	57	23.7	22	31	33	28
Vitamin C	32	17.8	47	19.5	8	11.3	23	20.3
Vitamin B	19	10.6	31	12.9	8	11.3	5	4.2
Vitamin E	18	10	43	17.8	16	22.5	9	7.6
**Herbal medications**								
Garlic	24	13.3	22	9.1	3	4.2	2	1.7
Celery	13	7.2	18	7.5	3	4.2	1	0.8
Gingko biloba	2	1.1	5	2.1	10	14.1	9	7.6
**Nutritional supplements**								
Cod liver oil	22	12.2	37	15.4	9	12.7	11	9.3
Fish oil	6	3.3	5	2.1	3	4.2	8	6.8
Glucosamine	0	0	0	0	4	5.6	10	8.5

The percentages are calculated over the number of participants that reported use of at least one CAM indicated as N

Analgesics were the most common class of OTC drugs at all waves (5.4 to 8.8%), followed by laxatives (2.0% to 5.1%) and antithrombotic agents (low dose aspirin) (1.6% to 2.6%). The most commonly used analgesic was paracetamol, while laxatives were mostly senna, docusate or a mixture of both.

### Variables affecting self-medication

OTC use was not associated with age, gender, education level and tertiary education in any of the four waves [Table 5]. Although there was a statistically significant association between income and OTC use in waves 3 and 6, the pattern of results was inconsistent for the different waves. The same was true for self-rated health.

**Table 5:** Prevalence of OTC use across variables of interest

	1992-1993		1994-1995		2000-2001		2003-2004	
	Wave 1	N = 268	Wave 3	N = 278	Wave 6	N = 79	Wave 7	N = 83
	
Variable	N	%	N	%	N	%	N	%
**Age (years)**								
65-79	159	13.0	148	16.6	27	12.5	8	17.0
80+	109	12.6	130	16.5	52	9.0	75	17.0
P value	0.809		0.985		0.149		0.997	
**Gender**								
Female	146	14.2	149	17.4	52	11.4	51	16.8
Male	122	11.6	129	15.6	27	8.1	32	17.4%
P value	0.075		0.318		0.121		0.873	
**Education** **(Age when left school)**								
<14 years	47	14.0	47	17.9	14	13.3	11	16.9
≥ 14 years	220	12.8	227	16.2	65	9.5	72	17.1
P value	0.538		0.493		0.223		0.965	
**Tertiary education**								
Yes	98	14.0	101	17.7	27	9.7	31	17.5
No	170	12.4	176	16.1	52	10.2	52	16.9
P value	0.316		0.388		0.843		0.859	
**Income Level**								
<$12,000	89	13.0	97	17.4	26	15.8	23	19.8
$12,000 - $30,000	137	12.7	111	15.4	26	8.3	24	16.4
>$30,000	25	15.5	27	30.0	5	7.5	8	12.5
P value	0.598		0.003		0.029		0.446	
**Perceived Health**								
Excellent	19	9.9	17	13.2	4	6.3	4	25.0
Very Good	63	10.5	58	14.1	25	13.8	13	15.3
Good	96	15.2	105	19.7	21	9.4	25	14.6
Fair	73	15.3	73	18.1	18	12.2	24	21.2
Poor	17	9.4	14	13.2	4	10.8	6	20.2
P value	0.018		0.097		0.438		0.536	

The percentages are calculated over the total number of participants in each category of the variable of interest

CAM use was associated with age and gender [Table 6]. The younger elderly people were more likely to use CAM than the older group (80 years and over) in all four waves. The difference in use remained constant over time, with those age 65 to 79 years old being 1.5 to 1.6 times more likely to use CAM than those aged 80 years or more. Females were also found to be more likely users of CAM in waves 1, 3 and 6 (the results were not statistically significant in wave 7). CAM use was not associated with education level, tertiary education, income level and self-rated health in any wave except in wave 7 where use of CAM was higher in participants with tertiary education.

**Table 6:** Prevalence of CAM use across variables of interest

	1992-1993		1994-1995		2000-2001		2003-2004	
	Wave 1	N = 180	Wave 3	N = 241	Wave 6	N = 71	Wave 7	N = 173
	
Variable	N	%	N	%	N	%	N	%
**Age (years)**								
65-79	122	10.0	152	17.0	27	12.5	17	36.2
80+	58	6.7	89	11.3	44	7.7	101	23.0
P value	0.009		0.001		0.034		0.044	
**Gender**								
Female	102	9.9	142	16.6	53	11.6	81	26.7
Male	78	7.4	99	12.0	18	5.4	37	20.1
P value	0.041		0.007		0.002		0.098	
**Education** **(Age when left school)**								
<14 years	30	8.9	39	14.9	10	9.5	16	24.6
≥ 14 years	149	8.6	200	14.3	61	8.9	102	24.3
P value	0.862		0.803		0.840		0.954	
**Tertiary education**								
Yes	63	9.0	92	16.1	27	9.7	56	31.6
No	116	8.5	149	13.6	44	8.6	62	20.1
P value	0.694		0.161		0.600		0.004	
**Income Level**								
<$12,000	52	7.6	82	14.7	18	10.9	33	28.4
$12,000 - $30,000	101	9.3	120	16.7	29	9.3	34	23.3
>$30,000	12	7.5	13	14.4	10	14.9	12	18.8
P value	0.385		0.581		0.385		0.326	
**Self-rated health**								
Excellent	16	8.4	17	13.2	12	18.8	7	43.8
Very Good	58	9.7	64	15.6	17	9.4	24	28.2
Good	56	8.8	90	16.9	23	10.3	43	25.1
Fair	38	8.0	53	13.2	11	7.5	23	20.4
Poor	12	6.6	11	10.4	5	13.5	6	20.0
P value	0.722		0.312		0.152		0.268	

The percentages are calculated over the total number of participants in each category of the variable of interest

### Reasons for using CAM and OTC medicines

The main reasons for using analgesics were headache and pain (Table 7). Low dose aspirin was reported to be used for blood thinning and/or stroke prevention by most participants (Table 8). However, some respondents reported unlikely reasons for use such as for lowering cholesterol levels, hypertension and for sleep.

**Table 7:** Reasons for use of analgesics

	1992-1993		1994-1995		2000-2001		2003-2004	
	Wave 1	N = 112	Wave 3	N = 127	Wave 6	N = 44	Wave 7	N = 43
	
Reasons of use of analgesics	N	%	N	%	N	%	N	%
Headache	35	31.8	41	32.3	5	11.4	6	14
Specific pain	19	17.3	16	12.6	4	9.1	5	11.6
General pain	18	16.4	26	20.5	25	56.8	22	51.2
Other	21	19.1	25	19.7	5	11.4	3	7.0
Arthritic pain	11	10.0	15	11.8	2	4.5	4	9.3
Multiple reasons	6	5.5	4	3.1	3	6.8	3	7.0
Missing	2		0		0		0	

The percentages are calculated over the number of participants that reported use of analgesics

**Table 8:** Reasons for use of antithrombotic agents

	1992-1993		1994-1995		2000-2001		2003-2004	
	Wave 1	N = 34	Wave 3	N = 43	Wave 6	N = 17	Wave 7	N = 10
	
Reasons of use of antithrombotic agents	N	%	N	%	N	%	N	%
Blood thinning &/or stroke prevention	27	87.0	36	85.7	16	94.1	6	60.0
Unlikely reasons	4	12.9	5	11.6	1	5.9	4	40.0
Other	0	0.0	1	2.3	0	0.0	0	0.0
Missing	3		1		0		0	

The percentages are calculated over the number of participants that reported use of antithrombotic agents

The most common reasons for use of multivitamins were supplementation and maintenance of general health (Table 9). Vitamin C was predominantly used to ward off colds, to boost the immune system and for general health maintenance across all four waves (Table 10).

**Table 9:** Reasons for use of multivitamins

	1992-1993		1994-1995		2000-2001		2003-2004	
	Wave 1	N = 40	Wave 3	N = 57	Wave 6	N = 22 N = 22	Wave 7	N = 33
	
Reasons of use of multivitamins	N	%^1^	N	%^1^	N	%^1^	N	%^1^
Supplement	19	47.5	22	38.6	1	4.5	8	24.2
General health	14	35.0	27	47.4	21	95.5	19	57.6
Energy boost or tiredness	4	10.0	3	5.3	0	0.0	1	3.0
Other^1^	3	7.5	5	8.8	0	0.0	5	15.1

The percentages are calculated over the number of participants that reported use of multivitamins

^1 ^Other reasons included nerves or stress, arthritis, cold prevention and immune stimulation and multiple reasons

**Table 10:** Reasons for use of vitamin C

	1992-1993		1994-1995		2000-2001		2003-2004	
	Wave 1	N = 32	Wave 3	N = 47	Wave 6	N = 8	Wave 7	N = 23
	
Reasons of use of Vitamin C	N	%	N	%	N	%	N	%
Cold prevention and immune stimulation	13	43.3	14	30.4	0	0.0	8	34.8
General health	7	23.3	18	39.1	8	100	8	34.8
Supplement	6	20.0	7	15.2	0	0.0	3	13.0
Other^1^	4	13.3	7	15.2	0	0.0	4	17.4
Missing	2		1		0		0	

The percentages are calculated over the number of participants that reported use of Vitamin C

^1 ^Other reasons included muscles or bones, energy boost or tiredness, blood or circulation, arthritis, cardiac or heart health and multiple reasons

## Discussion

Up to a third of older participants in the current study used either CAM or OTC medicines. The study differed from Australian studies conducted earlier in several ways [6,12-14]. Firstly, this study focused on a targeted population, namely elderly people aged 65 and older in South Australia. The study also sought to provide a broader insight into the self-medication practices of the elderly in examining use of both CAM and OTC medicines and reasons for use.

Fewer elderly people in our survey reported use of OTC medicines (10% to 17%) compared to a range of 31% to 97% in other surveys [15-19]. However, these surveys were carried out in the United States (US). The discrepancies observed may be explained by differences in the definition of OTC medicines and differences in subsidised accessibility to prescription medicines between Australia and the US. Non-prescription vitamins and minerals are considered as OTC medicines by the US Food and Drug Administration (FDA) and CAM by the TGA [11,20]. Furthermore, there is no national subsidisation scheme for pharmaceuticals in the US while in Australia, residents may prefer to get most medicines on prescription due to low co-payment fees. This is particularly relevant for medicines such as paracetamol which is widely used in both countries and which is available as a subsidised prescription medicine only in Australia.

The reported use of CAM (8.6% to 24.2%) in our study was lower than in previous Australian studies (37% to 58%) [6,14,21-23]. Several differences in the methods used may account for this variation. All but two of the previous studies included alternative services such as acupuncture, massage and chiropractic in their definition of CAM. The time interval over which participants reported has also varied. A 2004 survey in South Australia found 37% of people older than 65 years were CAM users but participants were asked whether they had used any CAM over the past year [6]. A study with a similar definition of use to this current study in terms of time interval and products surveyed reported 43% of CAM use [21]. However, the format of the interview where examples of each type of supplement were provided may have prompted more reports of CAM use [21]. A recent study in Iceland was conducted using similar methods to ALSA where simultaneous recording of herbal and dietary supplements (HADS) and prescription medicine use was carried out [24]. This study reported a prevalence of 80% of HADS use among their participants.

The top three classes of OTC drugs used by our respondents did not change between 1992 and 2004 and were similar to those described previously in the 1989-1990 Australian National Health Survey [25]. Analgesics were the most commonly used class of OTC medicines, predominantly paracetamol. Painful chronic conditions are prevalent in the elderly and therefore, many are amenable to treatment by OTC analgesics. Other international studies have also found analgesics to be the most commonly reported OTC class, though aspirin and non-steroidal anti-inflammatory drugs (NSAIDs) were more commonly used than paracetamol [7,15,18,19]. It is unclear whether in these studies all aspirin use was grouped under analgesics but in our study, we distinguished between aspirin used at low dose as an antiplatelet agent or at a higher dose as an analgesic agent based on the medicine strength and indication of use. Low dose aspirin was found to be the third most commonly reported class of OTC drugs in our study.

Vitamins and minerals, herbal medicines and nutritional supplements were the three most common classes of CAM reported across all waves. This is consistent with findings reported in other Australian studies [9,21,22]. Multivitamins accounted for about one-third of all CAM used across the waves, followed by Vitamins C, B and E in agreement with the results of previous studies [21-26]. The most common herbal product to be reported in this study was garlic, also in agreement with other studies [21,26], with gingko biloba, marketed for age-related memory impairment, gaining popularity in the latest waves. Cod liver oil was the most popular nutritional supplement across all waves, with glucosamine use emerging as a treatment for arthritis after 2001, consistent with Brownie's prediction in 2000 [21]. One reason for the more recent emergence of this product in the study may be the shift towards inclusion of this product as part of conventional care.

None of the demographic variables tested, age, gender, education, tertiary education, income level and self-rated health was found to be associated with OTC use. The relationship of age and OTC use is equivocal in the literature [7,16,27,28]. Greater OTC use was observed in females in the US studies that included multivitamin use [7,16,27,28]. Greater educational attainment and poorer self-rated health have been associated with higher use of OTC in some studies [15,16,27].

Female gender and a younger age were the only variables found to be associated with CAM use in our study, in agreement with previous studies [12,14,15,21,23,26,29]. Possible explanations for CAM use were not available in the data because ALSA participants were not asked about attitudinal or psychological factors involved in their choice of medicines. A literature review of variables reported to influence the use of complementary medicines by consumers suggested that the emergence of postmodern values provided the best explanation of consumers' interest in CAM[9]. Postmodern values involved "a new set of beliefs about nature, science, holistic medicine, rejection of authority, individual responsibility and consumerism" [9]. A recent survey of attitudes of Australian consumers to complementary medicines found that many consumers saw their CAM use as "natural" and part of a holistic view of health [30].

Reasons reported for using OTC medicines were consistent with the indications approved by the Australian medicines agency with the exception of low-dose aspirin for which between 5.9% and 40.0% of participants reported unlikely reasons. The most common reasons for use of multivitamins were supplementation and maintenance of general health. In a 2004 Australian study that examined beliefs in CAM users, most respondents had no specific medical reason for using CAM but believed they would help their general health. Promotion of general health and prevention of illness were also found the main variables driving the use of CAM in other international studies [9]. Some CAM could also be used for the treatment of symptoms such as glucosamine for arthritis.

There were some potential limitations to our study. Although there was an apparent overall increase of participants reported use of at least one CAM or OTC from 19.4% at baseline to 35.5% by 2004, we did not attempt to assess statistically the time trend given the large amount of missing data due to the high attrition level at wave 7. Furthermore, slight variations in medication data collection methods in waves 3 and 7, and a higher proportion of women in wave 7 could partly explain the higher use of CAM and OTC medicines observed at this wave. Although many of the ALSA participants were from non-English speaking backgrounds, fluent English was essential and thus, the findings may not be a true reflection of the use of CAM or OTC in non-English speaking elderly community in a multi-cultural country. Finally, but like most other studies, the data we gathered was self-reported. However, unlike other studies, respondents were asked to show the medicine containers, even so some medicines could have been overlooked. The reasons for use of CAM were categorized from self-reported reasons by consumers and the categories were set up to reflect as closely as possible the words used by the consumers themselves. Then, it is not possible to infer from this data whether use of CAM for "general health" or as "supplement" reflect different beliefs as regards the therapeutic effect of CAM. The question of whether consumers perceived themselves to be "deficient" in a vitamin or believe that supra-therapeutic doses would improve physical or mental performance cannot be determined from the data collected in the ALSA database.

This study focused on examining the types of non-prescription medications used by older Australians and the association between use and various demographic and health-related factors. Further work is needed to examine how self-medication amongst older people contributes to polypharmacy and increases the risk of adverse drug reactions. Use of NSAIDs and aspirin, for example, are associated with an increased risk of adverse drug events, hospitalization and death, with the elderly being particularly vulnerable [31]. Some OTC medicines may also have severe interactions with prescribed medicines [32].

## Conclusion

Self-medication among the ALSA respondents ranged from 18% to 36% between 1992 and 2004. The most frequent classes of CAM were vitamins and minerals, herbal medicines and nutritional supplements, with younger individuals and women more likely to use them. For OTC medicines, the most commonly used were analgesics, laxatives and low dose aspirin. Use of OTC medicines seemed to be done in accord with indications officially approved by the Australian medicine agency. Future work should examine risks associated with the concomitant use of CAM, prescription and OTC medicines.

## List of abbreviations

**ABS**: Australian Bureau of Statistics; **AIHW**: Australian Institute of Health and Welfare; **LSA**: Australian Longitudinal Study of Ageing; **ASMI**: Australian Self-Medication Industry; **ATC**: Anatomical Therapeutic Chemical; **CAM**: Complementary and Alternative Medicines; **FDA**: US Food and Drug Administration; **HADS**: Herbal and Dietary Supplements; **HIC**: Health Insurance Commission; **NPS**: National Prescribing Service; **NSAIDs**: Non-Steroidal Anti-Inflammatory Drugs; **OTC**: Over-The-Counter; **TGA**: Therapeutic Goods Administration.

## Competing interests

The authors declare that they have no competing interests.

## Authors' contributions

LYG wrote the research proposal, did the review of the literature, performed the analysis and wrote the first draft of the manuscript. AV and SS designed the study, participated in the analysis and the interpretation of the data and reviewed the manuscript. ML conducted the ALSA study, provided raw data and reviewed the manuscript. All authors read and approved the final manuscript.

## Pre-publication history

The pre-publication history for this paper can be accessed here:

http://www.biomedcentral.com/1472-6882/9/42/prepub
